# Targeting the Ubiquitin-Proteasome System for Cancer Therapeutics by Small-Molecule Inhibitors

**DOI:** 10.3390/cancers13123079

**Published:** 2021-06-20

**Authors:** Gabriel LaPlante, Wei Zhang

**Affiliations:** 1 Department of Molecular and Cellular Biology, College of Biological Science, University of Guelph, 50 Stone Rd E, Guelph, ON N1G2W1, Canada; glaplant@uoguelph.ca; 2 CIFAR Azrieli Global Scholars Program, Canadian Institute for Advanced Research, MaRS Centre West Tower, 661 University Avenue, Toronto, ON M5G1M1, Canada

**Keywords:** ubiquitin (Ub), ubiquitin-proteasome system (UPS), cancer, small-molecule, proteolysis-targeting chimera (PROTAC), E3 ligase, deubiquitinase (DUB)

## Abstract

**Simple Summary:**

The ubiquitin-proteasome system regulates multiple facets of protein homeostasis to modulate signal transduction in numerous biological processes. Not surprisingly, dysregulation of this delicately balanced system is frequently observed in cancer progression. In the past two decades, researchers in both academia and industry have made significant progress in developing small-molecule inhibitors targeting various components in the ubiquitin-proteasome system for cancer therapy. Here, we aim to provide a comprehensive summary of these efforts. Additionally, we overview the advancements of targeted protein degradation, a recently emerging drug discovery concept in cancer therapy.

**Abstract:**

The ubiquitin-proteasome system (UPS) is a critical regulator of cellular protein levels and activity. It is, therefore, not surprising that its dysregulation is implicated in numerous human diseases, including many types of cancer. Moreover, since cancer cells exhibit increased rates of protein turnover, their heightened dependence on the UPS makes it an attractive target for inhibition via targeted therapeutics. Indeed, the clinical application of proteasome inhibitors in treatment of multiple myeloma has been very successful, stimulating the development of small-molecule inhibitors targeting other UPS components. On the other hand, while the discovery of potent and selective chemical compounds can be both challenging and time consuming, the area of targeted protein degradation through utilization of the UPS machinery has seen promising developments in recent years. The repertoire of proteolysis-targeting chimeras (PROTACs), which employ E3 ligases for the degradation of cancer-related proteins via the proteasome, continues to grow. In this review, we will provide a thorough overview of small-molecule UPS inhibitors and highlight advancements in the development of targeted protein degradation strategies for cancer therapeutics.

## 1. Introduction

**Figure 1:**
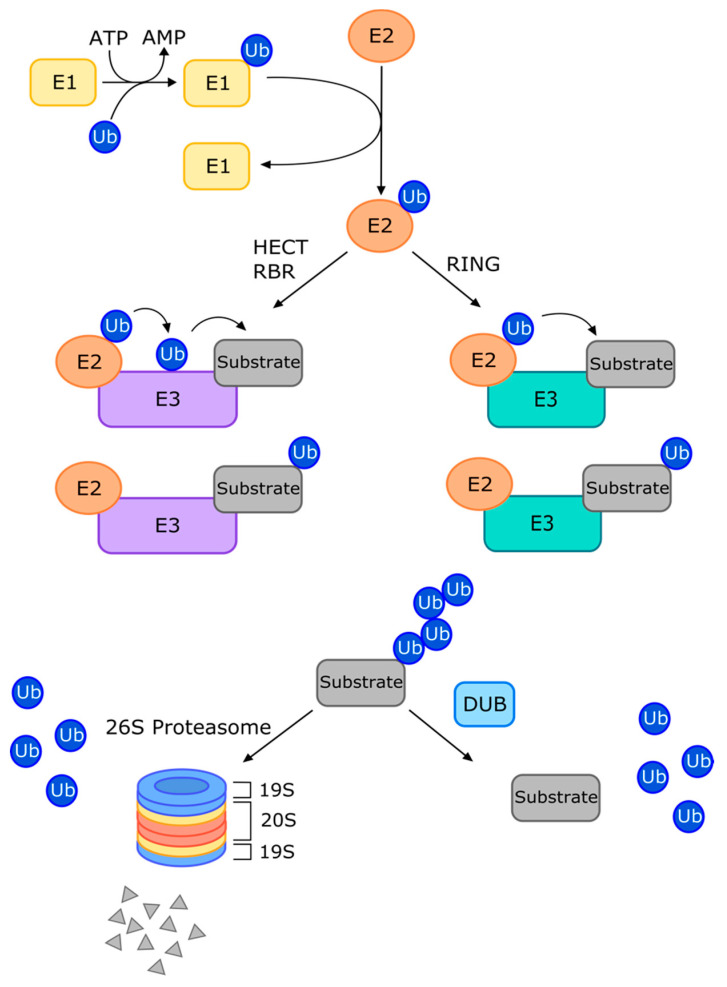
Overview of the ubiquitin-proteasome system (UPS). The E1, E2, E3 enzyme cascade results in the ubiquitination of a protein substrate. HECT and RBR-type E3 ligases perform a two-step ubiquitination, whereas RING E3s perform direct Ub transfer. K48-linked poly-ubiquitination results in proteasomal degradation, whereby the 19S proteasomal subunit removes Ub moieties prior to the substrate’s proteolytic degradation by the 26S subunit. DUBs can rescue substrate expression or alter cell signaling by removing Ub moieties. Ub, ubiquitin; HECT, Homologous to E6-AP carboxyl terminus; RBR, RING-between-RING; RING, really interesting new gene; DUB, deubiquitinase.

The ubiquitin-proteasome system (UPS) is vital for protein homeostasis and is implicated in many cellular processes, including DNA repair [1], endocytic trafficking [2], and the immune response [3]. The wide range of activities regulated by the UPS stems from the remarkable specificity and diversity of signal transduction events enabled by the process of ubiquitination. Ubiquitination is a post-translational protein modification involving the covalent attachment of ubiquitin (Ub), a highly conserved 76-amino acid polypeptide, to a substrate via the coordinated activities of a Ub-activating enzyme (E1), a Ub-conjugating enzyme (E2), and a Ub ligase (E3) (Figure 1) [4,5,6]. In the presence of ATP, Ub is activated by E1 before being delivered to the E2 enzyme, and this is followed by its eventual transfer to the substrate proteins, catalyzed by a E3 ligase [5,7]. Most commonly, Ub is reversibly conjugated either at a lysine (K) residue or an N-terminal amino acid and can be attached as a single polypeptide (mono-ubiquitination) or a polymer of at least four (poly-ubiquitination) [4,7,8,9,10]. Ub itself contains seven lysines, any of which can be an attachment site for the next molecule in a poly-Ub chain. Additional layers of complexity arise by post-translational modifications and the addition of Ub-like molecules, such as NEDD8, to Ub chains [11]. The resulting diverse possibilities of mixed, branched, or modified chains provides the basis for the functional diversity of Ub signaling.

The eventual fate of a ubiquitinated protein depends on the nature of the Ub chain. The most well-studied linkage is the K48-linked polyubiquitination, which generally targets proteins for proteasomal degradation [6]. The 26S proteasome is a large protein complex consisting of the 20S catalytic core and 19S regulatory subunit(s) [12]. The 19S subunit cleaves the Ub moiety from the protein substrate and threads the unfolded protein into the catalytic core, where the substrate is degraded via chymotrypsin-like, trypsin-like, and caspase-like proteolysis activities [10,12,13]. The second-best understood linkage, K63, has important non-proteolytic roles, including functions in protein trafficking [2,14] and the DNA damage response pathways [1]. Importantly, K48 chains can also be involved in non-proteolytic functions, while K63 chains, conversely, have known proteolytic functions [7]. The other five lysine linkages (K6, K11, K27, K29, and K33) are less well-studied, although this topic has recently been comprehensively reviewed [15]. Briefly, K6-linked poly-Ub chains have been shown to be involved in autophagy and the DNA damage response; K11 chains are implicated in cell cycle regulation and proteasomal degradation or stabilization; K27 chains are important actors in the innate immune response; K29 chains are involved in cell signaling, with potentially protective roles in neurodegenerative diseases; and K33 chains have purported roles in cell signaling and protein trafficking. The diverse arsenal of Ub signals implies that Ub-interacting proteins must possess some means of deciphering the code, and indeed, the eukaryotic genome contains an array of Ub binding domains (UBDs) that can confer linkage specificity to a Ub receptor [16]. While many UBDs are promiscuous on their own, the structural environment created between the UBD and its surrounding domains, whether on the same protein or adjacent proteins within a complex, can confer remarkable selectivity [7].

While the specific mechanism of ubiquitination can vary, the process is generally catalyzed by the E1→E2→E3 signaling cascade [5,7]. There are two known E1 activating enzymes, dozens of E2 enzymes, and over 600 E3 ligases encoded in the human genome [17]. The E3 ligases can be classified depending on their structural domains and mechanism of action into three major families, termed homologous to E6-AP carboxyl terminus (HECT), really interesting new gene (RING), and RING-between-RING (RBR) families [18]. Intriguingly, an additional accessory factor called an E4 enzyme can be involved in the lengthening of Ub chains [7]. On the other side of the spectrum, a class of enzymes called deubiquitinases (DUBs) oppose the action of the E3 ligases. DUBs can trim, edit, and remove Ub moieties from protein substrates, thereby preventing their degradation or altering cell signaling [19,20,21]. Around 100 DUB enzymes are encoded in the human genome, for which seven phylogenetically-delineated families exist: the Ub-specific proteases (USPs), Ub carboxyl-terminal hydrolases (UCHs), the otubain/ovarian-tumor-domain containing proteins (OTUs), the Machado–Joseph disease domain superfamily (MJDs), the JAB1/MPN/MOV34 proteases (JAMMs), the motif interacting with Ub-containing DUB family (MINDYs), and the zinc finger containing peptidase (ZUP1) family, which has one human representative [5,11,22]. Similar to other Ub-interacting proteins, DUBs contain UBDs that dictate their linkage specificity [22].

A harmonious balance between ubiquitination and deubiquitination is essential for protein homeostasis. Definitive links between aberrant Ub signaling and disease have been identified, and the misregulation of the UPS is a contributing factor for many types of cancers [13,23]. Evidence underscoring roles of the UPS in tumorigenesis, tumor metabolism, and tumor survival has been recently summarized [17]. For example, mutations in the E3 ligase F-box/WD repeat-containing protein 7 (FBXW7) can contribute to tumorigenesis through accumulation of its oncogenic targets including MYC and mTOR [24,25]. The UPS can ubiquitinate mitochondrial outer membrane proteins [26,27], turn over mitochondrial oxidative phosphorylation proteins [28], and modulate redox balance [29], all of which signal its role in regulating tumor metabolism [17]. Tumor survival is dependent on the UPS’ regulation of various pro-apoptotic pathways, including modulation of TNF signaling through RIP1 ubiquitination [17,30]. Additionally, cancer cells may particularly rely on the UPS for protein homeostasis and tumor survival because of their increased rates of protein turnover [5,31]. For all of these reasons, the UPS has been identified as an attractive target for development of cancer therapeutics. 

To date, the proteasome; E1, E2, E3; and DUBs have all been targeted for inhibition by small-molecule inhibitors. The early success achieved by proteasome inhibitors such as bortezomib [32] encouraged researchers to look for small-molecule inhibitors of the other UPS players. Over the past number of years, both traditional and innovative approaches to small-molecule screens have been employed for the identification of novel chemical inhibitors [33]. Another promising strategy exploiting the UPS is targeted protein degradation, such as the proteolysis-targeting chimera (PROTAC) technology [34]. PROTAC therapeutics can utilize small-molecule “warheads” to hijack UPS machinery and selectively target proteins of interest for proteasomal degradation via their ubiquitination, offering the promise of a greatly expanded scope of druggable targets within the human proteome. Here, this review will provide an updated overview of small-molecule inhibitors of the UPS, as well as developments in targeted protein degradation and PROTAC technology, in the context of cancer therapeutics.

## 2. Small-Molecule Inhibitors Targeting the UPS

### 2.1. Proteasome Inhibitors

The proteasome was the first UPS component to achieve clinical success as a therapeutic target [32]. The high rate of protein turnover in cancer cells, paired with the marked absence of proteasome abnormalities in human cancers, indicated that cancer cells may require increased use of the proteasome [5,35]. Subsequently, proteasome inhibitors (PIs) targeting the 20S catalytic core particle, such as bortezomib [12,32], carfilzomib [36,37], oprozomib [38], and ixazomib [39], or targeting the 19S regulatory particle, such as IU1 [40], b-AP15 [41], VLX1570 [42], RA-9 [43], WP1130 [44], and RA190 [45], have been widely developed as cancer therapies (Table 1).

Bortezomib is the first and most successful of the PIs used in clinics for cancer therapy. Bortezomib downregulates NF-κB signalling (activation of which has been implicated in the progression of many cancers) by reversibly binding the 20S subunit active site and preventing proteasomal degradation of inhibitors of NF-κB (IκBs) [12,35,46]. Bortezomib was also shown to inhibit NF-κB activation in multiple myeloma (MM) cells via inhibition of tumor necrosis factor alpha (TNF-α) [47]. Bortezomib has been approved by the U.S. Food and Drug Administration (FDA) for treatment of MM [32,48], mantle cell lymphoma (MCL), non-small-cell lung cancer (NSCLC), and pancreatic cancer [12,49,50,51,52,53]. However, similar to other PIs, its use is limited by adverse effects and drug resistance. For example, Bortezomib-induced peripheral neuropathy (BIPN) is a common limiting toxicity that can result in sensory alteration, motor weakness, and severe pain, sometimes permanently impairing the patient’s quality of life [54,55]. Furthermore, acquired Bortezomib resistance is relatively common, limiting its applicability. Two separate studies revealed resistance mechanisms hinging on acquired mutations in the proteasome β5-subunit (PSMB5) protein that impacted Bortezomib binding to the proteasome in Borteomib-resistant cell models [56,57]. Due to the broad nature of inhibition and the relatively common acquisition of resistance for PIs, targeting upstream UPS regulators such as the E1-E2-E3 enzyme cascade or DUB enzymes is an attractive alternative [58].

### 2.2. Targeting E1 and E2 Enzymes

There are only two inhibitors targeting the E1 activating step in the E1-E2-E3 cascade: PYR-41 for the inhibition of UBA1 [90,91] and MLN4942 (i.e., Pevonedistat), a small-molecule inhibitor of NEDD8-activating enzyme (NAE) [81]. NEDD8 is a Ub-like protein whose conjugation to cullin-RING E3 ligases (CRLs) is imperative for the degradation of CRL substrates, many of which are relevant in various cancers [92]. The E1 enzyme NAE activates NEDD8, and Pevonedistat covalently binds NEDD8, forming a complex that prevents NAE’s conjugation of NEDD8 to CRL and causing accumulation of CRL substrates [81,93]. Pevonedistat is being investigated in several clinical trials, including two Phase 3 trials currently recruiting for treatment of patients with acute myelogenous leukemia (AML) (Table 1).

E2 enzymes confers significantly more potential targets for small-molecule inhibitors compared to the E1s. However, since E2 can associate with multiple E3s, less attention has been given to the E2s as drug targets compared to the E3s due to specificity concerns [17]. Nevertheless, CC0651 and NSC697923 (Table 1) are small molecules that inhibit human E2 enzymes and have shown some success at reducing cancer cell growth [88,89]. The vast number of available E3 and DUB enzymes and their characteristically substrate-specific mechanisms make them ideal downstream UPS targets for inhibition by cancer therapeutics, as described below.

### 2.3. Targeting E3 Ligases

As mentioned above, the E3 ligases can be divided into three main classes with different structures and catalytic mechanisms. The RING E3 ligases are defined by the RING domain that coordinates two Zinc ions at the structural level [94]. Mechanistically, the RING E3 ligases act as a scaffold to bring the E2–Ub complex and the substrate together and facilitate direct Ub transfer from E2 to substrate [95]. Within the RING E3 class, there is the CRL superfamily, which consists of a cullin protein (CUL) acting as a scaffold to interact with an adaptor and substrate receptor [96]. The CRLs are well represented by the Skp1-Cul1-F-box protein (SCF) E3s, in which one of the ~70 human F-box proteins can perform substrate recognition [97]. In contrast to the RING E3s, the HECT E3s performs a more catalytic role in ubiquitination by forming a thioester bond with Ub via a cysteine residue in the HECT domain before Ub transfer [98]. The 28 known human HECTs are divided into three families based on their N-term structure: NEDD4, HERC, and “other” [99]. The third major class of E3 ligases, the RBRs, consists of around 13 members in humans, defined by two RING domains separated by a cysteine-rich in-between-ring (IBR) domain [95]. RBR E3s employ a hybrid mechanism of the RING and HECT E3s, and well-known RBR members include PARKIN, HOIP, and HOIL-1L [95,100].

Due to the high substrate selectivity by which E3s mediate protein degradation, their inhibition poses little risk of off-target effects [10]. However, it is important to consider the context of the target tissue/tumor type, as E3 ligases can have tumor suppressing or promoting effects at the same time [17]. Inhibition of cancer promoting substrates without affecting normal cells is desired, but the complicated regulatory systems at play can pose challenges. To this end, many small-molecule inhibitors have been identified for human E3 ligases, with varying levels of success seen in vitro, in cells, in animal models, and in clinical trials (Table 2). 

#### 2.3.1. RING-Type E3 Ligases

##### Targeting the MDM2–p53 Interaction

**Figure 2:**
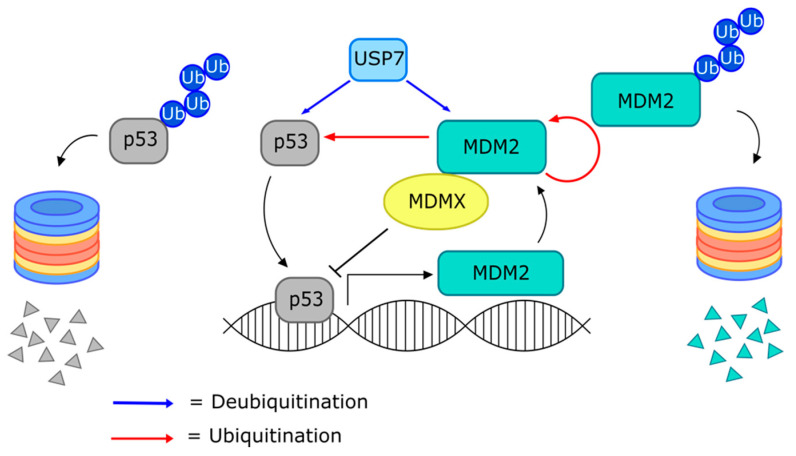
The USP7–MDM2–p53 axis. USP7 deubiquitinates both p53 and MDM2 but has stronger affinity for MDM2. MDM2 performs ubiquitination of p53 and self-ubiquitination. P53 promotes the expression of its target genes, including MDM2. MDMX can heterodimerize with MDM2 to facilitate p53 ubiquitination or directly block p53 action.

MDM2 (murine double minute 2) negatively regulates protein and transcriptional levels of the tumor-suppressive p53 (Figure 2) [196,197] and, therefore, is frequently observed as mutated or overexpressed in many cancers [198]. Thus, it was hypothesized that inhibition of MDM2-mediated p53 degradation could impair tumor formation and growth, and several small-molecules have been identified to impede the p53–MDM2 interaction [199]. The Nutlins, discovered in 2004, are cis-imidazoline analogs which structurally mimic p53 to bind MDM2, thereby preventing their association [199]. Nutlin-3a is a particularly active member that has been reported to selectively induce p53-dependent apoptosis in glioblastoma and AML cells [101,108] and also induce p53-independent mechanisms of tumor cell death [200,201]. Several preclinical studies have shown promising results for Nutlin-3a in targeting various solid tumors and hematological malignancies, either as a single treatment or in combination with other drugs [102,103,105,106,107]. Subsequently, the optimized Nutlin derivative RG7112 (aka RO5045337) [109] became the first MDM2 inhibitor to advance to clinical trials. Phase 1 trials investigating RG7112 against MDM2-amplified liposarcoma [111], solid tumors [112], and leukemia [110] reported limited efficacies and high incidences of adverse events, especially hematological toxicities. RG7388 (aka RO5503781, Idasanutlin) was developed in response to the required high doses and the side-effects associated with RG7112 [113]. Recently, Phase 1 results were published reporting promising in vivo efficacy of RG7388 in patients with polycythemia vera (PV) and essential thrombocythemia (ET) [114] and AML [115], where the treatments were well-tolerated aside from low-grade gastrointestinal toxicities. However, the first Phase 3 study of Idasanutlin, investigated in combination with cytarabine to treat AML, was recently terminated based on interim efficacy results (study identifier NCT0254). 

Many other small-molecule inhibitors of the MDM2–p53 interaction have been developed, and several clinical trials have recently published (Table 2). MI-77301 (aka SAR405838) is a molecule arising from affinity optimization of spirooxindole for binding to MDM2, and preclinical studies showed its ability to impair cell growth in a p53-dependent manner in acute leukemia, osteosarcoma, prostate, and colon cancer cell lines [117]. MI-77301 was subjected to two clinical studies so far, with one suggesting safety and potential for use in combination treatments for advanced cancers, including solid tumors and lymphoma [118]. MK-8242 (aka SCH-900242) has been evaluated for treatment of AML [120] and advanced solid tumors [121], but limited efficacy was reported, and adverse events were common. AMG 232 has shown pre-clinical promise in vivo against various tumor xenografts and cell lines [123,124]. Clinical evaluation of AMG 232 for treatment of relapsed/refractory AML yielded promising results, despite some adverse effects noted at higher doses [125]. DS3032b is an MDM2 inhibitor that has been undergoing clinical evaluation for a number of years; two clinical trials were recently completed that have shown promising preliminary results [128,129], but no further updates are available so far. HDM201 is an imidazolopyrrolidinone analogue that inhibits the p53–MDM2 interaction [202] and is currently being subjected to clinical evaluation alone or in combination treatments against various solid or hematological malignancies. Preliminary results reported good bioavailability and induced tumor regression in cancer cell and xenograft animal models [131,132]. Other MDM2 inhibitors currently undergoing clinical studies include APG-115 [134], APR-246 [138], and CGM097 [136]. Inhibitors of the p53–MDM2 interaction in preclinical stages include MI-63 [141], MI-219 [142], and the natural product sempervirine [143].

Some small-molecule inhibitors have been developed by directly targeting p53. One such compound is RITA (reactivation of p53 and induction of tumor cell apoptosis), which binds p53 directly and results in its accumulation in tumor cells [144]. Syl-155 [145] and HLI373 [146], on the other hand, are molecules that bind MDM2 in a competitive manner with p53 to cause accumulation. MDMX (murine double minute X) is another protein implicated in p53 regulation that can either bind p53 directly, blocking its transcriptional activity, or heterodimerize with MDM2 and facilitate p53 ubiquitination [203,204] (Figure 2). There is a need for dual inhibitors of MDM2/MDMX as it is highly expressed in some cancers [205,206]. NSC207895 [148] and MEL24 [147] are small molecules that target MDMX and have been shown to activate p53 and induce tumor cell apoptosis.

##### CRL/SCF RING E3 Ligases

The SCF complexes are members of the CRL family of RING E3 ligases [97,203]. The variable F-box proteins (FBPs) are responsible for SCF substrate specificity and are often overexpressed in cancers or correlated with poor prognosis [204,205]. Drugs targeting components of various cancer-related SCFs, including FBXW7, SKP2, FBXO3, FBXL3, and *β*-TrCP1, have been developed [203]. Orodonin is a natural compound that promotes FBW7-mediated proteasomal degradation of the oncogenic c-MYC, inducing apoptosis in leukemia and lymphoma cells in preclinical studies [149]. SCF-I2 binds the substrate-interacting cleft of FBW7, allosterically inhibiting Cdc4 recognition, thereby impeding tumor progression in colon and prostate cancer models [150]. SMER3, a specific inhibitor of the FBP Met30 [206], was identified via screening for small molecules capable of enhancing rapamycin, which inhibits the activity of TOR (target of rapamycin), a protein kinase often overexpressed in human cancers [207,208,209]. SCF-SKP2 is thought to elicit oncogenic effects by promoting degradation of various tumor suppressors, including p27 [152,204]. Compound A [151]; SMIP004 [152]; and the compounds C1, C2, and C3 [153] are small-molecule SKP2 inhibitors that have been shown to stabilize p27 in various preclinical cancer models. Many other molecules have reduced SKP2 expression in cancer cells, including SZL-P1-41 [154], longikaurin A [155], curcumin [156,157], and other natural compounds [156,210,211,212,213]. SCF-*β*-TrCP1 can induce proteasomal degradation of the inhibitor of nuclear factor-κB (IκB) via ubiquitination, resulting in overexpression of NF-κB signaling and tumorigenesis [214,215,216]. Inhibition of *β*-TrCP1 has been achieved by erioflorin, resulting in stabilization of tumor suppressor genes and decreased cancer cell proliferation [161]. GS143 operates similarly, inhibiting *β*-TrCP1-mediated IκB ubiquitination and suppressing NF-κB signaling [217].

##### Other RING E3s

RNF4 is a RING-type E3 ligase that promotes degradation of SUMOylated proteins via their ubiquitination [218]. An activity-based chemical library was screened against RNF4, and TRH 1-23 was identified as a binder with low inhibitory activity [162]. Using this ligand as a starting point, the analog CCW-16 was generated as a more potent RNF4 inhibitor. Subsequent addition of a linker to the BET bromodomain family inhibitor JQ1 generated CCW 28-3, a bifunctional degrader of RNF4, and interestingly, the final product showed even higher binding affinity for RNF4 than the CCW-16 ligand alone.

Inhibitor-of-apoptosis proteins (IAPs) inhibit apoptosis via suppression of caspase activity [219]. IAPs such as XIAP, cIAP1, and cIAP2 possess RING-finger domains and E3 ligase activity and are often overexpressed in many cancers [163,219]. Small-molecule mimetics of the IAP-inhibiting Smac proteins have been found to activate caspase and induce TNFα signaling [220]. GDC-0152 is a Smac mimetic that showed promising preclinical results in inhibiting tumor growth and inducing apoptosis of tumor cells [163], but clinical trials were terminated before proceeding from Phase I. Other IAP inhibitors that entered clinical trials include LCL161, AT-406, AEG 35156, AEG 40826, TL 32711, and YM155 [221]. Published results report that weekly oral doses of LCL161 was well-tolerated and efficacious in treating patients with advanced solid tumors, supporting further investigation [167]. Two publications have arisen from clinical evaluation of AT-406 (aka DEBIO1143, SM-406) against metastatic solid tumors [171] and head and neck cancers [172]. The recent Phase 2 study concluded that the addition of AT-406 augmented standard-of-care therapy with manageable safety, warranting Phase 3 investigation [172].

The tumor necrosis factor receptor-associated factor (TRAF) family of proteins are well-known as adaptor proteins important in cell signaling [222,223,224], but most TRAF proteins contain a RING domain, and some can act as E3 ligases [224,225]. TRAF6 is a RING E3 ligase whose overexpression has been implicated in lung cancer and osteosarcoma cells [226,227,228]. The binding of TRAF6 to the E2 enzyme Ubc13 results in the generation of K63-linked Ub chains that spur immune responses via NF-κB signaling [179]. Researchers identified the molecule C25-140 through a high-throughput small-molecule screening approach and found it inhibits the TRAF6–Ubc13 interaction with high selectivity [179]. The molecule did not inhibit the action of other RING-type E3 ligases, nor tested HECT E3s. Through a different approach, researchers developed a series of molecules that decrease levels of TRAF6 [181]. BC-1215 is a small-molecule antagonist of FBXO3, which inhibits the degradation of Fbc12, which in turn, results in destabilization of TRAF6 and reduces cytokine-driven inflammation in mice [181].

#### 2.3.2. HECT-Type E3 Ligase Inhibitors

HECT E3 ligases are associated with many types of human cancers, but discovery of small-molecule inhibitors for these targets has been limited, partly due to the poorly-characterized binding surfaces of HECT E3s [229]. WWP2 (WW domain-containing E3 Ub protein ligase 2) is a NEDD4-family HECT E3 ligase that can cause degradation of tumor suppressor proteins PTEN and Oct4 in many cancers [230]. Researchers identified five potential small-molecule inhibitors through high-throughput screening (HTS) of a chemical library, representing the first generation of WWP2 inhibitors [230]. SMURF1 (SMAD Ub regulatory factor 1) is another NEDD4 HECT that targets tumor suppressor RhoB for degradation [182,231]. HS-152 is a small-molecule inhibitor discovered via a cell-based HTS approach that blocks the catalytic activity of SMURF1′s HECT domains, stabilizing RhoB [182].

HUWE1 (HECT, UBA, and WWE domain-containing protein 1) is a regulator of several cancer-associated proteins, including MYC, p53, and MCL1, and its dysregulation or mutation is implicated in tumorigenesis [232]. Two relatively selective small-molecule inhibitors of HUWE1, BI8622 and BI8626, were identified via HTS and found to inhibit the growth of various colon cancer cell lines in vitro [183].

E6AP (E6-associated protein) is a HECT with well-documented roles in human papillomavirus (HPV)- and hepatitis C virus (HCV)-induced cancers. For example, E6AP can be hijacked by viral E6 protein to target p53 and/or retinoblastoma protein (Rb) for proteasomal degradation, contributing to the development of malignancies [229]. Some small-molecule inhibitors targeting the E6AP–p53 interaction have been identified, such as compound 12 [185], which was shown to rescue p53 function and inhibit proliferation of HPV infected cells. Luteolin and CAF024 are two flavonoid compounds that may prevent the hijacking of E6AP by binding to the hydrophobic binding groove of the viral E6 protein and preventing its association with E6AP [186]. Recently, a rational in silico screening strategy yielded three promising small-molecule inhibitors, termed Lig1, Lig2, and Lig3 [233]. N-acetyl phenylalanine prevents the trimerization of E6AP and inhibits its E3 functionality, albeit at high concentrations [229,234]. Finally, CM11-1 is a macrocyclic N-methyl peptide inhibitor that has been shown to prevent the polyubiquitination of Prx1 and p53 by E6AP in a RaPID cell-free system [235].

Using a phage display method, bicyclic peptide inhibitors of HECT domains were identified that prevented E2 binding by SMURF2, WWP1, and other HECT E3 ligases [236]. Researchers screened for small molecules capable of displacing one of the peptides from SMURF2 and discovered a compound, termed Heclin (HECT ligase inhibitor), that caused a conformational change in the HECT domain to prevent its activity. Heclin indiscriminately inhibited HECT ligases in vitro and killed growing HEK293 cells, but the approach could potentially be used to find more specific inhibitors.

#### 2.3.3. RBR-Type E3 Ligase Inhibitors

RBRs are the least frequently targeted class of E3 ligases for inhibitor development so far. The RBR structures, mechanisms, and relevance to cancer are less well-understood compared to the RING and HECT E3s, and the topic has been recently reviewed [237]. 

LUBAC (linear Ub chain assembly complex), consisting of HOIP, HOIL-1L, and SHARPIN, is the only E3 ligase known to perform polyubiquitination via Met1 linkages [238,239,240,241]. LUBAC activity and mutation is implicated in various diseases, including activated B cell-like diffuse large B cell lymphoma (ABC-DLBCL) [242]. BAY11-7082 [187], gliotoxin [243], and HOIPIN-8 [189] are three compounds reported to inhibit LUBAC. Bendamustine, an FDA-approved chemotherapeutic for leukemia and lymphoma, has been classified as an alkylating agent but was also found to selectively inhibit HOIP [244]. Three other small molecules that displayed promising selectivity for HOIP were identified via a covalent ligand screen [245]. Finally, the recent development of a strategy using single-domain antibodies (dAbs) shows promise for expeditious rational design of inhibitors for RBRs [246]. Tsai et al. (2020) screened a synthetic library of dAbs to obtain tight binders of the RBR domain of HIOP and solved the co-crystal structure of one complex to demonstrate the platform’s amenability to ligand-soaking [246].

### 2.4. Chemical Inhibitors of DUBs

DUBs reverse the action of the E3 ligases by editing or removing Ub chains via their proteolytic activity [19,20,21]. Thus, inhibiting DUBs can promote the selective degradation of proteins by preventing the removal of their Ub tags. The target specificities and defined catalytic sites of DUBs make them easy to screen against small-molecule libraries and therefore became attractive therapeutic targets [247,248]. Multiple small-molecule DUB inhibitors have been reported, albeit with limited selectivity until recently [58]. Below, we summarize the discovery of inhibitors for the most popular DUB target, USP7, and highlight recent progress in the development of other DUB inhibitors (Table 3).

#### 2.4.1. USP7 Inhibitors

The largest family of DUBs, the USPs, are characterized by a catalytic USP domain of variable size containing six conserved motifs [247]. USP7 deubiquitinates various substrates implicated in numerous cellular processes, but its important and paradoxical role in the p53–MDM3 axis has made it a popular target for cancer therapeutics [249]. On one hand, USP7 deubiquitinates and stabilizes p53, reversing the action of MDM2 [250]. However, USP7 can also stabilize MDM2 by opposing its auto-ubiquitination. Interestingly, when cellular USP7 levels are decreased, the net result is destabilized p53, but when USP7 activity is abolished, MDM2 is downregulated, stabilizing p53 [251] (Figure 2). Thus, discovery of potent small-molecule USP7 inhibitors is a long sought-after avenue for cancer therapeutics.

The first generation of small-molecule USP7 inhibitors included HBX 41,108; HBX 19,818; HBX 25,258; P5091; P50429; and P22077. HBX 41,108 was the first inhibitor of USP7 to be identified. It uncompetitively and reversibly inhibited USP7 but had poor selectivity [249]. HBX 19,818 was later shown to covalently bind the active site of USP7, selectively and irreversibly inhibiting its activity, resulting in increased p53 levels in human cancer cells, inducing apoptosis in a dose-dependent manner [252]. HBX 28,258 also inhibited USP7 by the same mechanism. Another group discovered P5091, which is selective for USP7, which caused apoptosis of Bortezomib-resistant MM cells and prolonged survival in mouse models [253]. P5091 is, in fact, a dual inhibitor of USP7 and USP47 [254]. Optimization of P5091 led to P50429 [254] and P22077 [255,256], which bind irreversibly to the catalytic cysteine C223 of USP7. However, none of these “first-generation” USP7 inhibitors had optimal potency or specificity [257,258], perhaps as a result of their activity-based discovery approach.

Almost a decade later, several potent and selective allosteric USP7 inhibitors were identified by different groups simultaneously through combination of fragment-based screens and structure-guided medicinal chemistry optimization. This strategy is likely to have general applicability for targeting other USP DUBs in the future. Gavory and colleagues first identified “Compound 1” through small-molecule fragment-based screening utilizing surface plasmon resonance (SPR), followed by specific modifications based on features of other known USP7 inhibitors [259]. Subsequent medicinal chemistry-based design produced “Compound 4”, which noncompetitively binds an allosteric site distal to USP7′s catalytic site. Compound 4 exhibited ~2000-fold increased potency over Compound 1, showed good cell permeability, and various cancer cells were found to be hypersensitive to the compound [259]. The allosteric inhibition mechanism could be part of the reason for Compound 4′s dramatic potency and selectivity [267], and it might exploit mechanistic features of USP7 related to the conformational changes that occurred following Ub binding [258]. Another group similarly utilized NMR-based screening, followed by structure-based design, to generate the allosteric modulators GNE-6640 and GNE-6776 [268]. The compounds prevented Ub from interacting with USP7 by binding its catalytic domain at a novel functional site 12 Å away from the catalytic cysteine residue [268]. They showed potent inhibition with high selectivity for USP7 and proved capable of inducing tumor cell death [260,268]. Lamberto et al. used structure-based design to optimize a previously identified small-molecule that had high specificity for USP7 but weak potency [261]. XL188 non-covalently inhibited the active site of USP7 with 100-fold higher potency than the original lead and was shown to increase the expression of p53 and of downstream tumor suppressor p21, resulting in impaired tumor growth [261]. Finally, Turnbull et al. (2017) reported FT671 and FT827 as specific allosteric inhibitors of USP7 with high affinity exhibited in vitro and in human cells [262]. In an approach similar to other groups, a small-molecule library was screened for binding and inhibitory activity against USP7, followed by structure-guided optimization. FT671 exhibited in-cell results consistent with successful USP7 inhibition and compromised tumor growth in a p53-dependent manner in a mouse model [262].

#### 2.4.2. Other DUB Inhibitors

USP1 is a DUB whose catalytic activity is stimulated upon complex formation with UAF1 [269]. One of its targets is FANCD2, which has essential functions in the DNA repair pathway [270]. As a pharmacological target, inhibition of the USP1/UAF1 complex was proposed to impair tumorigenesis in malignant osteosarcoma and sensitize cells to chemotherapy [248]. Two small molecules, pimozide and GW7647, were identified in 2011 [263] and found to non-competitively inhibit USP1 and inhibit proliferation of cisplatin-resistant NSCLC cells in combination treatment with cisplatin.

USP14 is one of three proteasome-associated DUBs that perform their deubiquitination activities at the proteasome and are important therapeutic targets for chemical inhibition [247]. USP14 reversibly associates with the proteasome and inhibits the degradation of proteasome-targeted proteins via trimming of their Ub chains [40], and its dysregulation has been implicated in tumorigenesis and neurodegenerative disorders [58]. IU1 is a selective inhibitor of USP14, identified via a small-molecule HTS strategy, that was shown to promote the degradation of proteins, including the neurodegenerative disease-associated proteins Tau and ataxin-3 [40]. IU1-47 is one of 87 variants of IU1 developed by Boselli et al. (2017), with 10-fold higher potency and retained selectivity for USP14 inhibition [271]. Another IU1 analog, 1B10, also has heightened potency, as well as better membrane permeability [272]. IU1-248 was also recently discovered to be around 10-fold more potent than IU1 [72]. Interestingly, structural analysis revealed that this family of molecules functions by binding a previously unknown steric site on USP14. b-AP15 is an inhibitor of USP14 and another proteasome-associated DUB, UCHL5 [73]. b-AP15 selectively blocks 19S regulatory particle DUB activity and has been shown to induce tumor cell apoptosis in various solid tumor types, as well as MM [41,73,74,273]. VLX1570 is an optimized lead of b-AP15 that also inhibits USP14 and UCHL5 [42], but limiting toxicities terminated clinical trials (study identifier NCT02372240). Rpn11 is the third proteasome-associated DUB [274]. Several small-molecule inhibitors of Rpn11 have been identified, including 8TQ and Capzimin [79,275]. Non-specific inhibitors of Rpn11 include SOP11, an epidithiodiketopiperazine molecule [80], and thiolutin, a naturally-occurring antibiotic [276], which both also inhibit other JAMM family DUBs.

Recently, UCHL1 became a popular target for inhibition in many cancers, as well as neurodegenerative diseases and liver and lung fibrosis [58]. Interestingly, Ott et al. (2017) utilized a fluorometric cell lysate-based assay called AlphaLisa to develop UCHL1 inhibitors as proof-of-principle for this strategy, yielding a series of inhibitory compounds, the most selective being celasterol and mangaferin [277]. LDN-57444 is an isatin O-acyl oxime molecule identified through HTS and medicinal chemistry that selectively inhibits UCHL1 [278]. Both LDN-57444 and its soluble version LDN-Pox exhibited anti-metastatic effects against oral squamous cell carcinoma (OSCC) cell line [264]. Finally, two selective inhibitors of UCHL1 called 6RK73 and 8RK64 were recently synthesized for the purpose of developing activity-based probes to monitor UCHL1 activity in cell systems [279].

USP9X is a DUB that stabilizes the oncogenic MCL-1 in cancer cells [280] and was shown to be overexpressed in MM [281]. WP1130 [282] is a DUB inhibitor with some selectivity for USP9X that was shown to reduce MCL-1 and induce cancer cell apoptosis, as well as reduce chemoresistance in various tumor types [44,75,283]. However, upregulation of USP24 in response to USP9X knockdown, promoting myeloma cell survival, demonstrated the need for dual USP9X/USP24 inhibitors, prompting the development of EOAI3402143 (aka G9) by improving the properties of WP1130 [265]. Both WP1130 and G9 also partially inhibit another DUB, USP5, but the resultant p53 accumulation only enhances anti-tumor effects in MM [265]. Recent preclinical results have shown WP1130 and G9 to be effective at inducing apoptosis in other cancers, such as AML [78] and melanoma [76]. More recently, FT709 [266] was identified as a potent inhibitor of USP9X, with greatly extremely improved specificity compared to WP1130. 

OTUB2 (otubain-2) has been shown to be associated with various human diseases [284,285,286], including cancer, in which its deubiquitination of transcriptional regulators YAP and TAZ promotes metastasis [287]. OTUB2 preferentially cleaves Lys63-linked poly-Ub chains but also has activity against Lys11- and Lys48-linked chains [288]. Recently, an electrophile-fragment screening approach was used to discover the first covalent inhibitor for OTUB2, termed OTUB2-COV-1 [289]. STAMBP is another Lys63-specific DUB, for which the small molecule BC-1471 was reported to be a potent and specific inhibitor [290]. Finally, many broad-spectrum DUB inhibitors, such as chalcone molecules G5 and F6 [291], PR-619 [256], and betulanic acid [292] have been discovered, but unspecific inhibition of DUBs can result in undesirable off-target effects and high toxicities, making them unideal for clinical application [293].

## 3. Targeted Protein Degradation

The chemical compounds described above fit the classical pharmacological model, where small molecules bind an active or allosteric site of a protein target to interfere with its catalytic function. While traditionally this is a reasonable approach, it has been pointed out that this model limits the druggable protein space, because less than 25% of the human proteome consists of proteins that can be targeted in this way [294,295,296,297,298,299]. Moreover, the so-called “occupancy-driven” approach requires high drug concentrations for inhibition, leading to unwanted toxicities and off-target effects [297,298,300]. A promising alternative strategy is targeting natural protein–protein interactions (PPIs) instead of focusing on ligand-binding sites [33]. The shift to an “event-driven” pharmacological model will expand the druggable space to include a much wider spectrum of proteins involved in critical cellular functions and pathologies [33,297,300]. By exploiting UPS machinery to capitalize on transient PPIs, selective degradation of protein targets can be achieved. Indeed, proteolysis-targeting-chimeras (PROTACs) are rapidly emerging as a technology holding great promise for cancer treatment [296,298,300,301,302,303].

### 3.1. PROTACs

**Figure 3:**
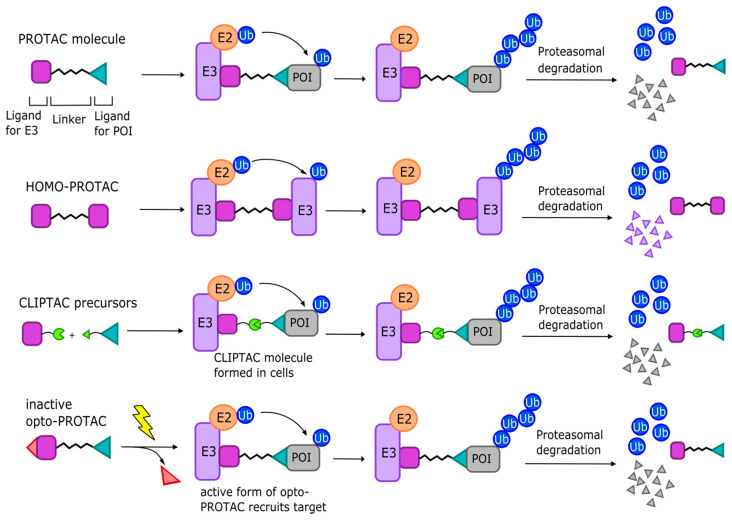
Schematic of the mode of action of PROTAC and selected variations. PROTAC (proteolysis-targeting chimera) molecules consist of a ligand for recruiting an E3 ligase and a ligand for recruiting a POI connected by a linker. PROTAC molecules and their variations aim to orient the POI in proximity to the E3 ligase to facilitate its ubiquitination and proteasomal degradation, after which the PROTAC can be used again. Homo-PROTACs contain two identical E3-recruiting moieties to perform E3 ubiquitination. CLIPTACs consist of cell-permeable precursors that join together via click-chemistry to form an active molecule in cells. Opto-PROTACs, photo-PROTACs, or PHOTACs are activated by UVA or visible light to recruit their targets in cells.

A proteolysis-targeting chimaera (PROTAC) is a bivalent molecule comprising two selective binding moieties (termed “warheads”) to recruit a protein target and an E3 ligase, which are linked together so that the target will be brought to close proximity of the E3 ligase for its ubiquitination (Figure 3) [298,301]. PROTACs have been shown in vitro and in vivo to deplete target proteins in a rapid and sustained fashion, providing a significant advantage versus small-molecule-based protein inhibition, where compensatory responses can increase target concentrations over time [304,305]. Other noteworthy advantages of PROTACs are their high selectivity, their ability to bypass the challenge of drug resistance due to mutated active sites, and the fact that the toolkit of possible warheads is vast due to the ability to use agonists, antagonists, or non-modulatory binders of the target at hand [306]. Finally, since PROTACs degrade rather than inhibit their targets, they are effective even at very low concentrations (in some cases 1000 times lower than traditional drugs) [302]. In the following sections, we will briefly summarize the history of PROTAC optimization and highlight recent developments and considerations.

#### 3.1.1. First Generation PROTACs

Early PROTACs were peptide-based, which had limitations due to instability, poor cell permeability, and low activity [300]. The first PROTAC was developed by Sakamoto et al. (2001) and used a peptidic recruiter of the E3 ligase SCF-β-TRCP [301]. IκBα is a natural target of SCF-β-TRCP, and its recruitment is mediated by a phosphorylated 10-aa sequence within IκBα, DRHDSGLDSM [307]. Sakamoto and colleagues exploited this IκBα phosphopeptide (IPP) as a SCF-β-TRCP ligand in the pioneering PROTAC study [301]. PROTAC-1, as it was termed, consisted of the IPP warhead linked to ovacilin, a covalent binder of MetAP-2. While it did induce degradation of MetAP in Xenopus extracts, it was not cell permeable, and its application was limited by instability of the molecule. Subsequently, PROTACs targeting the nuclear receptors AR and ER were developed but still required microinjection to function in cells [308]. In 2004, a PROTAC was developed that was capable of permeating cells due to the inclusion of a poly-D-arginine cell penetrating peptide [309]. Schneekloth et al. (2004) linked peptidic ligands for the E3 VHL and the target FK506 binding protein (FKBP), and the resulting PROTAC degraded FKBP in cells [309].

#### 3.1.2. Development of Small-Molecule PROTACs

The second generation of PROTACs employed small-molecule warheads instead of peptides, first exemplified by the use of Nutlin-3a to recruit MDM2 and a selective androgen receptor modulator (SARM) ligand to target the androgen receptor (AR) [310]. This PROTAC’s activity was still limited, requiring micromolar concentrations, but it partially degraded AR in cells. Researchers continued to search for ways to decrease size and employ novel E3 ligase recruiters for new PROTACs. The discovery of the pthalimide immunomodulatory drugs (IMiDs) as binders of cereblon (CRBN) in 2010 [311], shortly followed by the optimization of potent synthetic ligands for VHL, provided new tools for PROTAC development and significantly advanced the field forward [312,313,314,315].

The small-molecule VHL ligands were first utilized by Buckley et al. (2015) to degrade HaloTag7 (HT7)-GFP fusion proteins [316]. Incorporation of the VHL ligand in a so-called HaloPROTAC, along with a chloroalkane linker that binds HT7, resulted in GFP fluorescence depletion in cells. Bondeson et al. (2015) employed the VHL ligand to create PROTACs targeting estrogen-related receptor alpha (ERRα) and the serine-threonine kinase RIPK2, and their activities were proven by the depletion of ERRα in breast cancer cells and depletion of RIPK2 in monocytes [302]. Furthermore, this study showed the first successful in vivo PROTAC results by the reduction (~40%) of ERRα in mouse tumors following intraperitoneal injection. Another study [317] used a VHL ligand-based PROTAC to degrade the bromo- and extra-terminal (BET) protein BRD4, which has been strongly linked to cancers, including AML and ovarian carcinoma [318,319,320]. By linking the VHL ligand to the BET inhibitor JQ1, the compound MZ1 was created, which induces selective degradation of BRD4 over BRD2 and BRD3 in cells [317]. Raina et al. (2016) also linked JQ1 to VHL ligand to produce the potent ARF-771, with high efficacy against castration-resistant prostate cancer (CRPC) models, showing the first successful application of PROTACs against solid tumor types [321].

Around the same time in 2015, two groups reported PROTACs employing IMiDs to recruit CRBN. The BRD4 inhibitor OTX015 was linked to pomalidomide to generate ARV-825, which reduced BRD4 in a superior manner to OTX015 alone in lymphoma cell lines [322]. Winter et al. (2015) utilized JQ1 tethered to a thalidomide derivative to produce dBET1, which reduced BET proteins, induced apoptosis of AML and lymphoma cells, and delayed leukemia progression in a mouse model [323]. Interestingly, by using only a different linker compared to the Zengerle et al. (2015) study [317], no preferential selectivity for BRD4 was observed by Winter et al. (2015), suggesting the importance of linker geometry in ternary complex formation and target specificity [323].

Recent progress in the field of small-molecule PROTACs has been thoroughly reviewed elsewhere [300,303,324,325,326]. While CRBN [327,328,329,330,331,332,333,334,335,336,337,338,339] and VHL [340,341,342,343,344,345,346,347,348,349,350] ligands are still by far the most utilized to recruit E3 ligases in PROTACs, other E3 ligases have been employed in small-molecule PROTACs, including MDM2 [351,352,353], KEAP1 [354], DCAF16 [355], RNF4 [162], and RNF114 [356]. It is noteworthy that two PROTACs, ARV-110 and ARV-471, entered clinical trials recently (study identifiers NCT03888612 and NCT04072952). These PROTACs, developed by Arvinas LLC, have proprietary structures, but ARV-110 targets AR, ARV-471 targets ERα, and both have shown successful degradation of their targets in cell lines and mouse models for prostate cancer and breast cancer, respectively [357,358].

#### 3.1.3. PROTAC Variations

Another class of PROTAC-like molecules is the specific and non-genetic IAP-dependent protein erasers (SNIPERs). The first of these capitalized on the discovery that methyl bestatin (MeBS) interacts with cIAP1 and induces its autoubiquitination [359]. Researchers added a ligand for the target protein, cellular retinoic acid binding proteins (CRABPs) I and II, to the methyl residue of MeBS. Consequently, the first SNIPERs induced the simultaneous degradation of the recruited E3 and the target proteins [359]. This could be useful in targeting cancer cells where IAP E3 ligases are overexpressed [360]. However, in further developments of SNIPERs, researchers replaced an ester group with an amide group at the linker attachment site of MeBS to circumvent the autoubiquitination [361]. Subsequently, many SNIPERs have been developed with various target protein ligands, such as ERα [362,363], AR [364], and BRD4 [365]. We noted that SNIPERs have been reviewed extensively elsewhere [360].

Other variations of PROTACs have been developed as well (Figure 3). Homo-PROTACs hijack E3 ligases to induce their self-degradation [366,367,368]. The first of these was developed by the Ciulli group in 2017 and induced the dimerization and auto-degradation of VHL, potentially serving as a useful research tool [366]. Another PROTAC variation, called CLIPTACs, or in-cell click-formed proteolysis targeting chimeras, were developed in an attempt to overcome the cell permeability issue [369]. CLIPTACs assemble intracellularly by click-chemistry following cell treatment with cell-permeable precursors. In 2016, this approach proved successful in the development of CLIPTACs that degraded protein targets BRD4 and ERK1/2 [369]. Interestingly, recent research has developed PROTACs that can be activated by UV or visible light, termed photo-PROTACs, opto-PROTACs, or PHOTACs [370,371,372,373]. These could be applied clinically to directly target tumors via photodynamic therapy (PDT), providing an encouraging new development for the field of photomedicine.

#### 3.1.4. PROTAC Future Directions

Careful consideration of the design and optimization of PROTACs for clinical safety and efficacy has been thoroughly reviewed [300,374,375,376,377]. It is notable that the stability of the ternary complex (target-PROTAC-E3) is potentially more important than the binary affinities of each warhead for efficient target degradation [300,302]. The favorable or repulsive interactions between PROTAC components and the substrate protein lead to positive or negative cooperativity, which in turn influence the stability of the ternary complex [300,378]. For example, the crystal structure of a BRD4-degrading PROTAC’s ternary complex revealed cooperativity via van der Waals contacts between BRD4 and both the VHL warhead and the linker, which heightened the PROTAC’s specificity and potency [340]. While, historically, the linker design received much less attention, it is now becoming increasingly clear that linker composition is one of the most important factors contributing to PROTAC cellular activity, as linker length and attachment location can influence ternary complex conformation, target selectivity, and cell permeability [297,302,379]. Further advancement in computational analysis and structure-based optimization methods will likely result in better PROTAC linker design. A more extensive overview of the different linker classes, effects on the multiple aspects of PROTACs properties, and emerging design principles can be found here [380,381].

Expanding the repertoire of PROTAC warheads will enable the recruitment of more E3 ligases and the degradation of more protein targets. There is opportunity to utilize low affinity molecules or resurrect discarded “near-drugs”, optimized binders that failed clinical trials, for inclusion as PROTAC warheads [297]. Screening small-molecule libraries for binders of target proteins without active sites will allow PROTACs to engage more diverse protein types. Protein engineering strategies could also be employed to develop warheads against E3 ligases or target proteins that have no small-molecule binders to date. Expanding the toolbox of E3 ligases will be critical for future PROTAC development, as only a handful of the over 600 human E3 ligases have been employed so far [377]. When choosing an E3 ligase, an important consideration is that it must be present in the target tissue or cell type for the PROTAC to be active, adding complexity as well as presenting an opportunity for tissue-specific applications [377]. For example, there are four human E3 ligases currently known to be expressed in specific tissues that also induce proteasomal degradation of protein targets. Recruitment of these in PROTACs against various cancer-associated targets could mediate tissue-specific degradation without effecting normal cells in other tissues [377]. As progress in the field of PROTACs continues to be made, care must be taken to investigate the efficacy, specificity, and pharmacological properties of each newly developed drug. Expansion of in vivo data and available pharmacokinetic and pharmacodynamic analysis models will be essential to bring PROTACs closer to clinical application [376].

### 3.2. Other Notable Approaches to Targeted Protein Modulation

The IMiDs, mentioned above as CRBN ligands in PROTACs, are successful mediators of targeted protein degradation in their own right. As “molecular glues”, they can bring the CRL4–CRBN E3 ligase complex together with substrates including Ikaros, Aiolos and CK1α to mediate their degradation [311,382,383]. Thalidomide, and its derivatives lenalidomide and pomalidomide, are FDA-approved for use against various hematological malignancies [311,383,384,385]. Other molecules currently undergoing clinical studies are avadomide and iberdomide [386,387]. Indisulam, tasisulam, and chloroquinoxaline sulfonamide (CQS, aka NSC 339004) are aryl sulfonamide molecules that induce the association of nuclear protein RBM39 with the E3 ligase complex Cul4–DCAF15, inducing its proteasomal degradation in a manner similar to the action of IMiDs [388]. Clinical trials using these molecules for the treatment of solid tumors have been performed, but none of them advanced due to limited efficacy. However, it has since been suggested that the molecules be revisited for the treatment of leukemias and lymphomas due to these cells’ heightened dependence on RMB39 [388].

Hydrophobic tagging is another strategy for targeted protein degradation. Fulvestrant is a therapeutic that exposes a hydrophobic patch on the surface of ERα and thereby mediates its degradation [389]. Inspired by this modality, hydrophobic tagging mimics a partially unfolded protein state to induce proteasomal degradation [34]. In 2014, the previously “undruggable” target Her3 was potently degraded in cells by a bivalent molecule (TX2-121-1) derived from coupling a covalent ligand to a hydrophobic adamantyl moiety [390]. Similarly, selective degradation of the AR was induced by a molecule consisting of the hydrophobic adamantyl moiety and known AR ligand RU59063 [391], adding to the cohort of existing selective androgen receptor downregulators (SARDs).

Other means have also been pursued to achieve targeted protein degradation. Chimeric proteins similar to PROTACs have been developed, in which a CHIP E3 ligase was linked to either the scFv of an antibody or a fibronectin type III domain monobody; and these molecules successfully depleted protein targets in cells [392]. However, the disulfide bonds present in the scFvs caused challenges in achieving optimal folding within the cell cytoplasm. Finally, selective degradation of proteins was achieved by using a Boc3Arg moiety linked to small-molecule inhibitors of the protein targets; the mode of action involved no ubiquitination, instead relying on direct interaction between the Boc3Arg compound and the proteasome [393].

Finally, DUBs can be recruited for targeted protein stabilization. In the first ever reported study of targeted deubiquitination, Kanner et al. developed engineered DUBs (enDUBs) to facilitate Ub removal and rescue of target proteins [394]. The first-in-class enDUBs consisted of the catalytic domains of OTUD1 fused to a nanobody specific for their target. In their study, the researchers used enDUBs to rescue Ub-responsive mutations in deadly ion channelopathies, long QT syndrome (LQT), and cystic fibrosis (CF), but the enDUB technology could theoretically be applied to directly stabilize protective proteins in cancers. This idea opens the door for targeted protein deubiquitination by other means, for example, in a PROTAC-like molecule that recruits a DUB instead of an E3 ligase endogenously. As further developments in the strategy of targeted deubiquitination arise, they could provide a promising new avenue for therapeutic development.

## 4. Conclusions

The UPS is a rich resource of attractive targets for developing cancer therapeutics due to its essential role in controlling protein homeostasis. In cancer cells, precision targeting of UPS proteins via small-molecule inhibitors has been successful in affecting disease outcomes. However, the three main strategies for modulating the UPS (inhibiting the proteasome, inhibiting the E1-E2-E3 cascade, or inhibiting DUBs) are limited due to the important and complex roles served by each of these players. Unwanted effects of global modulation of individual UPS components can limit the utility of inhibitory molecules. Furthermore, the pharmacological model of inhibition by small molecules applies only to those members with well-defined active sites and catalytic functions, greatly limiting the druggable proteome. Thus, the emerging philosophy of targeted protein degradation, exemplified by the PROTAC technology, allows more fine-tuned control of pathological targets in cancer and other diseases.

Aside from the small-molecule strategies mentioned in this review, protein-based modulators (both activators and inhibitors) of UPS members have been widely developed. As an example, clinical trials are currently ongoing for a stapled α-helical peptide-based dual inhibitor of MDM2 and MDMX [395]. In addition, the structure-based combinatorial Ub variant (UbV) protein engineering technology leverages Ub as a scaffold to develop synthetic binders targeting Ub-interacting proteins, including those not easily targeted by small molecules [396]. UbVs have been developed so far to potently and specifically modulate DUBs [397,398,399], E2 enzymes [397,400], and E3 ligases [401]. The UbV platform has proven to be a robust research tool already, and its further development may represent an exciting future avenue in precision medicine. For example, UbVs can be used to develop small-molecule modulators through displacement screens or could be incorporated into targeted protein degradation applications, such as PROTACs [401].

Improving the specificity and efficacy of therapeutics by working towards tissue- and tumor-specific target degradation will improve treatment outcomes. As different approaches to screening technologies reveal new small-molecule inhibitors and ligands for targeted degradation methods, the field of precision medicine in cancer will benefit. Advancements in PROTACs, UbVs, enDUBs, and the other strategies mentioned in this review are examples of this paradigm moving closer to clinical reality.

## Figures and Tables

**Table 1:** Small-molecule inhibitors of the proteasome, E1 activating enzymes, and E2 conjugating enzymes.

Compound Name	UPS Target	Mode of Action	Current Clinical Stage	Cancer Models Targeted in Preclinical Studies	Targeted Cancer Types in Clinical Trials	Clinical Trial Results
**Proteasome Inhibitors**						
Bortezomib	Proteasome (20S particle)	Reversibly binds active sites of 20S proteasome	FDA approved (Phase 4)	MM, lymphocytic leukemia cells, oral squamous carcinoma cells, and more [32]	MM, AML, colorectal cancer, head and neck cancer, mantle cell lymphoma, and more	[59,60,61]
Carfilzomib	Proteasome (20S particle)	Irreversibly binds active sites of 20S proteasome	FDA approved (Phase 4)	Hematologic tumors [37]	MM, lymphoma, thyroid cancer, lung cancer, kidney cancer, and more	[36,62,63,64]
Oprozomib	Proteasome (20S particle)	Irreversibly binds active sites of 20S proteasome	Phase 1b/2	MM [65]	Solid tumors, hepatocellular carcinoma, MM, waldenstrom macroglobulinemia, and more	[66]
Ixazomib	Proteasome (20S particle)	Inhibits chymotrypsin-like activity of 20S proteasome	FDA approved (Phase 4)	Solid and hematologic tumors [67], MM [68], B-cell, plasma cell malignancies [69]	MM, relapsed/refractory MM, AML, Hodgkin and T-cell lymphoma, breast cancer, and more	[70,71]
IU1, IU1-47, 1B10, IU1-248	Proteasome (19S particle)	Inhibit USP14 (proteasome-associated DUB) via steric site	Preclinical	N/A	N/A	N/A
b-AP15	Proteasome (19S particle)	Targets UCHL5 and USP14 (proteasome-associated DUBs)	Preclinical	Solid tumors, MM [40,72,73,74]	N/A	N/A
VLX1570	Proteasome (19S particle)	Analog of b-AP15, also targets UCHL5 and USP14	Preclinical (Phase 1/2 terminated)	MM [42]	MM	N/A
WP1130	Proteasome (19S particle)	Inhibits USP9X, UCHL, and USP14	Preclinical	Chronic myelogenous leukemia (CML), melanoma, glioblastoma, myeproliferative disorders [44,75,76], MCL [77], AML [78]	N/A	N/A
RA-9	Proteasome (19S particle)	Inhibits proteasome-associated DUBs	Preclinical	Ovarian cancer [43]	N/A	N/A
RA190	Proteasome (19S particle)	Inhibits RNP13 and inactivates Uch37	Preclinical	MM, ovarian cancer [45]	N/A	N/A
Capzimin, 8TQ	Proteasome (19S particle)	Specifically target RPN11	Preclinical	Leukemia, NSCLC, breast cancer [79]	N/A	N/A
Thiolutin, SOP11	Proteasome (19S particle)	Targets RPN11 and other JAMM-family DUBs	Preclinical	Colon cancer, bortezomib-resistant RPE cells [80]	N/A	N/A
**E1 inhibitors**						
PYR-41	UBA1	Irreversibly binds active site cysteine of UBA1	Preclinical	N/A	N/A	N/A
MLN4942 (Pevonedistat)	NAE	Binds NEDD8, prevents CRL neddylation to inhibit activity	Phase 3	Colon cancer, lung cancer, myeloma, lymphoma [81,82,83]	AML, MM, lymphoma, melanoma, lung cancer, PCM, and more	[84,85,86,87]
**E2 inhibitors**						
CC0651	hCdc34	Binds allosteric pocket causing structural displacement	Preclinical	Prostate and colorectal cancer cell lines [88]	N/A	N/A
NSC697923	Ubc-Uev1A	Impedes formation of Ubc13 and Ub thioester conjugate	Preclinical	Diffuse large B-cell lymphoma (DLBCL) [89]	N/A	N/A

**Table 2:** Small-molecule inhibitors of the E3 ubiquitin ligases.

Compound Name	UPS Target	Mode of Action	Current Clinical Stage	Cancer Models Targeted in Preclinical Studies	Targeted Cancer Types in Clinical Trials	Published Clinical Trial Results
**MDM2 inhibitors**						
Nutlin-3	MDM2	Competitive inhibitor of p53 binding site on MDM2	Preclinical	AML [101], hematologic malignancies [102,103,104,105], breast cancers [106,107], glioblastoma [108]	N/A	N/A
RG7112 (RO5045337)	MDM2	Nutlin derivative, inhibits MDM2–p53 binding site	Phase 1	Cancer cell lines expressing wild-type p53 [109]	Leukemia, hematologic neoplasms, liposarcoma, advanced solid tumors, myeloproliferative neoplasms	[110,111,112]
RG7388 (RO5503781, Idasanutlin)	MDM2	Nutlin derivative, inhibits MDM2–p53 binding site	Phase 2 (Phase 3 terminated)	Cancer cell lines expressing wild-type p53, osteosarcoma xenografts [113]	AML, ALL, solid tumors, neuroblastoma, plasma cell myeloma, breast cancer, PV and ET, and more	[114,115,116]
MI-77301 (SAR405838)	MDM2	Selectively binds MDM2–p53 binding site, improved affinity via MDM2 N-term refolding	Phase 1	Leukemia, osteosarcoma, prostate and colon cancer cell lines [117]	Malignant neoplasms, advanced solid tumors, lymphoma	[118,119]
MK-8242 (SCH-900242)	MDM2	Oral MDM2–p53 inhibitor	Phase 1	N/A	AML, advanced solid tumors	[120,121]
AMG 232	MDM2	Inhibits MDM2–p53 interaction with improved potency due to hydrophobic interactions with MDM2 “glycine shelf”	Phase 1	Various tumor cell lines and xenografts [122,123,124]	AML, advanced solid tumors, glioblastoma, gliosarcoma, metastatic melanoma, MM, and more	[125,126]
Ds3032b (Milademetan)	MDM2	Oral MDM2–p53 inhibitor	Phase 2	Neuroblastoma [127], BCL [10]	Myeloma, AML, recurrent/refractory myeloid leukemia, advanced solid tumors, lymphomas	[128,129]
HDM 201 (Siremadlin)	MDM2	Binds to MDM2, inhibits interaction with p53	Phase 2	Wild-type-p53 cancer cell lines [130]	AML, colorectal cancer, liposarcoma, malignant solid tumors, and more	[131,132]
APG-115	MDM2	Oral MDM2–p53 inhibitor	Phase 2	Osteosarcoma xenografts [133]	AML, T-prolymphocytic leukemia, liposarcoma, advanced solid tumors, melanoma, salivary gland cancer	[134,135]
CGM097	MDM2	MDM2–p53 inhibitor	Phase 1	Colorectal cancer, osteosarcoma cells [136]	Solid tumors with wild-type p53	N/A
PRIMA1, APR-246	MDM2	Binds core domain of p53, preventing MDM2 association	Phase 3	Osteosarcoma, NSCLC, adenocarcinoma cell lines and xenografts [137,138], myeloma [139]	AML, myeloid malignancies, NSCLC, gastric cancer, esophageal carcinoma, non-Hodgkin’s lymphoma, CLL, MCL, hematologic neoplasms, and more	[140]
MI-63	MDM2	Binds MDM2, preventing p53 association	Preclinical	Prostate cancer cells with wt-p53 [141]	N/A	N/A
MI-219	MDM2	Binds MDM2, preventing p53 association; can also induce degradation of MDMX.	Preclinical	Various solid cancer cell lines, osteosarcoma xenografts [142]	N/A	N/A
Sempervirine	MDM2	MDM2–p53 inhibitor	Preclinical	Wt-p53 mouse embryonic fibroblast cell model [143]	N/A	N/A
RITA	MDM2	Binds wt-p53, preventing association with MDM2	Preclinical	Fibrosarcoma and colon cancer cell lines and wt-p53 tumor xenografts [144]	N/A	N/A
Syl-155	MDM2	Competitively inhibits MDM2–p53 binding	Preclinical	Wt-p53 fibrosarcoma cell line [145]	N/A	N/A
HLI373	MDM2	Competitively inhibits MDM2–p53 binding	Preclinical	Colon carcinoma cell line, wt-p53 transformed MEF cell model [146]	N/A	N/A
MEL24	MDMX/MDM2	Inhibits E3 ligase activity of MDM2–MDMX complex	Preclinical	Various wt-p53 cancer cell lines [147]	N/A	N/A
NSC207895 (XI-006)	MDMX	Represses MDMX transcription, activates p53 pathway	Preclinical	Various solid tumor cell lines [148]	N/A	N/A
**CRL/SCF RING E3 inhibitors**						
Oridonin	FBW7	Promotes proteasomal degradation of c-Myc via FBW7 agonism	Preclinical	Leukemia and lymphoma cell lines [149]	N/A	N/A
SCF-I2	FBW7	Blocks substrate-binding pocket, inhibits Cdc4, prevents substrate recognition	Preclinical	Colon and prostate cancer cell lines [150]	N/A	N/A
SMER3	Met30	Directly binds Met30, preventing association with SCF complex	Preclinical	N/A	N/A	N/A
Compound A	SKP2	Prevents association of SKP2 with SCF complex, results in accumulation of p27	Preclinical	MM cell lines, primary hematological malignancy cells [151]	N/A	N/A
SMIP004	SKP2	Downregulates SKP2, stabilizes p27	Preclinical	Prostate adenocarcinoma cell lines [152]	N/A	N/A
C1, C2, C3	SKP2	Sterically inhibits SKP2–Cks1–p27 interface	Preclinical	Metastatic melanoma cell lines, breast cancer cells [153]	N/A	N/A
SZL-P1-41 (Compound #25)	SKP2	Directly binds SKP2 to inhibit E3 activity	Preclinical	Prostate, lung, liver, and osteosarcoma tumor cell lines [154]	N/A	N/A
Longikaurin A	SKP2	Downregulates SKP2 expression	Preclinical	Hepatocellular carcinoma [155]	N/A	N/A
Curcumin	SKP2	Downregulates SKP2	Phase 3	Breast cancers [156], pancreatic cancer [157], glioma cells [158]	Prostate cancer, pancreatic cancer, colorectal cancer, MM, gastric cancer, breast cancer, leukemias and lymphomas, and more	[159,160]
Erioflorin	β-TrCP1	Interferes with β-TrCP1 to stabilize tumor suppressor Pdcd4	Preclinical	Kidney, breast, ovarian, and colon cancer cell lines [161]	N/A	N/A
GS143	β-TrCP1	Inhibits β-TrCP1 ubiquitination of IkB, suppresses NF-kB signaling	Preclinical	N/A	N/A	N/A
**Other RING E3 inhibitors**						
CCW 28-3	RNF4	Binds RNF4 to facilitate degradation of BRD4	Preclinical	Breast cancer cells [162]	N/A	N/A
GDC-0152	IAPs	SMAC mimetic, induces IAP degradation, activates caspases	Preclinical (Phase 1 terminated)	Breast cancer cell lines and xenografts [163]	Solid cancers	N/A
LCL161	IAPs	SMAC mimetic, induces degradation of cIAP-1	Phase 2	Various solid tumor cell lines [164], hepatocellular carcinoma [165], osteosarcoma [166], and more	Neoplasms, plasma cell myeloma, metastatic pancreatic cancer, myelofibrosis, small cell lung cancer, ovarian cancer, breast cancer, MM, and more	[167]
AT-406 (DEBIO1143, SM-406)	IAPs	SMAC mimetic; binds XIAP, cIAP-1, and cIAP-2; and activates caspases	Phase 3	Breast cancer [168], colorectal cancer [169], ovarian cancer [170]	Lymphoma, solid tumors, AML, NSCLC, squamous cell carcinoma of head and neck, MM, and more	[171,172]
AEG 35,156 (GEM640), AEG 40826	XIAP	Antisense oligonucleotides targeting XIAP mRNA to lower apoptotic threshold of cancer cells	Phase 2	Various cancer cell lines, xenograft models of colon, breast and osteosarcoma tumors [173]	Advanced solid tumors, leukemia, mammary carcinoma, pancreatic carcinoma, BCL, NSCLC, hepatocellular carcinoma, and more	[174]
TL 32711	IAPs	SMAC mimetic, induces degradation of cIAP-1, and caspase activation	Phase 2	MM cell lines and animal models [175]	Chronic myelomonocytic leukemia, relapsed epithelial ovarian cancer, myelodysplastic syndrome, peritoneal neoplasms, and more	[176]
YM155 (sepantronium bromide)	IAPs	Inhibits promoter of survivin gene (IAP protein)	Phase 2	Prostate cancer cell lines and xenografts [177]	Prostate cancer, melanoma, non-Hodgkin’s lymphoma, breast cancer, diffuse large-cell lymphoma, refractory B-cell lymphoma, and more	[178]
C25-140	TRAF6	Inhibits TRAF6–Ubc13 interaction specifically	Preclinical	Only studied in autoimmune and inflammatory disease models [179]	N/A	N/A
BC-1215	TRAF6 (via FBXO3 inhibition)	Antagonist of FBXO3, destabilizes TRAF6	Preclinical	Only studied in autoimmune and inflammatory disease models [180,181]	N/A	N/A
**HECT E3 ligase inhibitors**						
HS-152	SMURF1	Reversibly blocks SMURF1-mediated RHOB ubiquitination	Preclinical	Inhibited protrusive RHOB-dependent activity in cell lines [182]	N/A	N/A
BI8622 and BI8626	HUWE1	Inhibit HUWE1 to stabilize assembly of Myc-repressive MIZ1 complex on Myc-activated target genes	Preclinical	Colorectal cancer [183], MM [184]	N/A	N/A
Compound 12	E6AP	Inhibits oncogenic E6–p53 interaction in E6–E6AP–p53 complex	Preclinical	HPV-positive cervical carcinoma cell lines [185]	N/A	N/A
Lutolein and CAF024	E6AP	Bind viral E6 to prevent hijacking of E6AP	Preclinical	HPV-positive cervical carcinoma cells [186]	N/A	N/A
Lig1, Lig2, Lig3	E6AP	Inhibit E6–E6AP interaction	Preclinical	N/A	N/A	N/A
N-acetyl phenylalanine	E6AP	Dissociates active E6AP trimer	Preclinical	N/A	N/A	N/A
CM11-1	E6AP	E6AP inhibitor, prevents polyubiquitination of Prx1 in E6-independent and -dependent manner	Preclinical	N/A	N/A	N/A
Heclin	HECT, non-specific	Induces conformational change in HECT domain to inhibit activity	Preclinical	N/A	N/A	N/A
**RBR E3 ligase inhibitors**						
BAY 11-7082	LUBAC	Covalently binds active cysteine residues of E2′s Ubc13 and UbcH7 to prevent Ub conjugation	Preclinical	B-cell lymphoma, leukemia [187], gastric cancer [188]	N/A	N/A
gliotoxin	LUBAC	Selectively binds RBR domain of HOIP to inhibit Ub chain formation	Preclinical	N/A	N/A	N/A
HOIPIN-8	LUBAC	Inhibits LUBAC activity and suppresses NF-kB activation	Preclinical	ABC-DLBCL [189,190]	N/A	N/A
Bendamustine	LUBAC	Specifically inhibits HOIP	FDA approved (Phase 4)	Chronic lymphocytic leukemia, MM, non-Hodgkin’s lymphoma [191], and more	Ovarian cancer, MM, relapsed T-cell lymphoma, non-Hodgkin’s lymphoma, Hodgkin’s lymphoma, and more	[192,193,194,195]

**Table 3:** Small-molecule inhibitors of the deubiquitinases (DUBs).

Compound Name	UPS Target	Mode of Action	Current Clinical Stage	Cancer Models Targeted in Preclinical Studies	Clinical Trial Cancer Targets
HBX 41108, HBX 19818, HBX 28258	USP7	Covalently binds USP7 active site, increasing p53	Preclinical	Colon cancer cell lines [249,252]	N/A
P5091	USP7 and USP47	Dual inhibitor of USP7 and USP47	Preclinical	MM cells, bortezomib-resistant MM, and B-cell leukemia mouse models [253]	N/A
P50429, P22077	USP7	Irreversibly bind catalytic cysteine residue of USP7	Preclinical	Colon cancer cell line [254], neuroblastoma xenograft [255]	N/A
Compound 4	USP7	Selectively binds USP7 allosteric site	Preclinical	Leukemia, prostate adenocarcinoma cell lines [259]	N/A
GNE-6640, GNE-6776	USP7	Binds novel USP7 functional site	Preclinical	Colon cancer cells [260]	N/A
XL188	USP7	Non-covalent inhibition of USP7 active site, causing accumulation of p53 and p21	Preclinical	Breast cancer and MM cell lines [261]	N/A
FT671, FT827	USP7	Allosteric USP7 inhibition	Preclinical	Colon cancer, osteosarcoma cell lines, MM cells, and xenografts [262]	N/A
Pimozide and GW7647	USP1/UAP1 complex	Non-competitive inhibition of USP1	Preclinical	NSCLC cells [263]	N/A
IU1, IU1-47, 1B10, IU1-248	USP14	Binds USP14 steric site	Preclinical	N/A	N/A
b-AP15	USP14 and UCHL5	Blocks 19S DUB activity	Preclinical	Solid tumors, MM [40,72,73,74]	N/A
VLX1570	USP14 and UCHL5	b-AP15 analog, also blocks 19S DUBs	Preclinical (Phase 1/2 terminated)	MM [42]	Multiple Myeloma (MM)
Capzimin, 8TQ	Rpn11	Specific inhibitors of Rpn11	Preclinical	Leukemia, NSCLC, breast cancer [79]	N/A
Thiolutin, SOP11	Rpn11 (nonspecific)	Non-specifically inhibits JAMM-family DUBs	Preclinical	Colon cancer, bortezomib-resistant RPE cells [80]	N/A
Celasterol, mangaferin	UCHL1	Reversible inhibitors of UCHL1	Preclinical	N/A	N/A
LDN-57444, LDN-Pox	UCHL1	Incorporation of LDN-57444 in Pox micelle delivery system inhibits UCHL1 in cells	Preclinical	Oral squamous cell carcinoma cell line [264]	N/A
6RK73 and 8RK64	UCHL1	Cell penetrable probes bind UCHL1 active site cysteine in activity-dependent manner	Preclinical	N/A	N/A
WP1130	USP9X	Inhibits USP9X, UCHL, and USP14 to a degree	Preclinical	CML, melanoma, glioblastoma, myeloproliferative disorders [44,75,76], MCL [77], AML [78]	N/A
EOAI3402143 (G9)	USP9X/USP24 (and partially USP5)	WP1130 analog	Preclinical	AML [78], MM [265], melanoma [76]	N/A
FT709	USP9X	Competitively binds USP9X active site	Preclinical	Colon cancer cell line [266]	N/A
OTUB2-COV-1	OTUB2	Covalent OTUB2 inhibitor	Preclinical	N/A	N/A
BC-1471	STAMBP	Inhibits STAMBP, prevents interaction with NALP7	Preclinical	N/A	N/A
